# Modeling Doxorubicin-Induced Cardiotoxicity in Human Pluripotent Stem Cell Derived-Cardiomyocytes

**DOI:** 10.1038/srep25333

**Published:** 2016-05-04

**Authors:** Agnes Maillet, Kim Tan, Xiaoran Chai, Singh N. Sadananda, Ashish Mehta, Jolene Ooi, Michael R. Hayden, Mahmoud A. Pouladi, Sujoy Ghosh, Winston Shim, Liam R. Brunham

**Affiliations:** 1 Translational Laboratory in Genetic Medicine, National University of Singapore and the Agency for Science Technology and Research (A*STAR), Singapore; 2 Center for Computational Biology, Duke-NUS Graduate Medical School, Singapore; 3 National Heart Research Institute, National Heart Centre Singapore, Singapore; 4 Cardiovascular Academic Clinical Program, DUKE-NUS Graduate Medical School, Singapore; 5 Department of Medical Genetics, Centre for Molecular Medicine and Therapeutics, University of British Columbia, Vancouver, Canada; 6 Department of Medicine, Yong Loo Lin School of Medicine, National University of Singapore, Singapore; 7 Program in Cardiovascular and Metabolic Disorders, Duke-NUS Graduate Medical School, Singapore; 8 Department of Medicine, Centre for Heart Lung Innovation, University of British Columbia, Vancouver, Canada

## Abstract

Doxorubicin is a highly efficacious anti-cancer drug but causes cardiotoxicity in many patients. The mechanisms of doxorubicin-induced cardiotoxicity (DIC) remain incompletely understood. We investigated the characteristics and molecular mechanisms of DIC in human pluripotent stem cell-derived cardiomyocytes (hPSC-CMs). We found that doxorubicin causes dose-dependent increases in apoptotic and necrotic cell death, reactive oxygen species production, mitochondrial dysfunction and increased intracellular calcium concentration. We characterized genome-wide changes in gene expression caused by doxorubicin using RNA-seq, as well as electrophysiological abnormalities caused by doxorubicin with multi-electrode array technology. Finally, we show that CRISPR-Cas9-mediated disruption of *TOP2B*, a gene implicated in DIC in mouse studies, significantly reduces the sensitivity of hPSC-CMs to doxorubicin-induced double stranded DNA breaks and cell death. These data establish a human cellular model of DIC that recapitulates many of the cardinal features of this adverse drug reaction and could enable screening for protective agents against DIC as well as assessment of genetic variants involved in doxorubicin response.

Doxorubicin and other anthracyclines are highly efficacious anti-cancer medications and are used to treat a broad spectrum of adult and childhood malignancies, including Hodgkin’s and non-Hodgkin’s lymphoma, neuroblastoma, soft tissue sarcomas and breast cancer. Doxorubicin is also one of the most cardiotoxic medications in clinical use. Doxorubicin-induced cardiotoxicity (DIC) includes acute atrial and ventricular arrhythmia, as well as chronic cardiomyopathy and heart failure^1^,^2^. Despite decades of study, the precise mechanisms of DIC remain elusive and we currently lack the ability to predict or prevent this adverse drug reaction (ADR) in individual patients.

DIC is dose-dependent and at cumulative doses of 450 to 500 mg/m^2^ the incidence of congestive heart failure is 4 to 5%, whereas at doses of 550 to 600 mg/m^2^ the incidence increases to 18%^3^. One major hypothesis regarding the mechanism of DIC is that doxorubicin causes increased production of reactive oxygen species (ROS), leading to damage to DNA, proteins and lipids, and ultimately causing death and dysfunction of cardiomyocytes^4^,^5^. Other proposed mechanisms include mitochondrial dysfunction and alterations of Ca^2+^ homeostasis^6^,^7^. More recently, studies in mice have suggested an important role for topoisomerase-II beta (Top2β) in the pathogenesis of DIC^5^.

A major barrier to studying DIC has been the lack of appropriate model systems. This relates to the fact that human cardiac tissue, the primary site of DIC, is largely inaccessible and cannot be maintained in tissue culture. In addition, animal models may not accurately recapitulate DIC because of inter-species differences in both drug metabolism and cardiac structure and function. In particular, significant differences between mouse and human cardiac system in terms of electrophysiology and contractile features limit the extrapolation of findings from studies in murine systems to humans^8^,^9^,^10^,^11^,^12^.

An ideal model system would possess both high human physiological relevance and potential for high throughput applications. Recently, human pluripotent stem cell-derived cardiomyocytes (hPSC-CMs) have emerged as a powerful tool to model cardiac toxicity in highly physiologically relevant human cells^13^,^14^,^15^,^16^. Human pluripotent stem cells (hPSC) are capable of self-renewal and, because of their capacity to differentiate into cell type derivatives of all three germ layers, are a powerful tool for disease modeling and drug screening. Highly efficient differentiation protocols for generating cardiomyocytes (CMs) from hPSCs have been described in the past decade^17^,^18^,^19^,^20^,^21^,^22^,^23^, and these cells express major human cardiac ion channels and sarcomeric proteins, suggesting that they may have high human physiological relevance^13^,^24^. Key advantages of hPSC-CMs for studying cardiac toxicity include a higher degree of homology with human compared to animal CMs, and potential for high throughput applications. The objective of this study was to investigate the characteristics and molecular mechanisms of DIC in a hPSC-CM model system.

## Results

### Generation and characterization of hPSC-derived CMs

**Figure 1:**
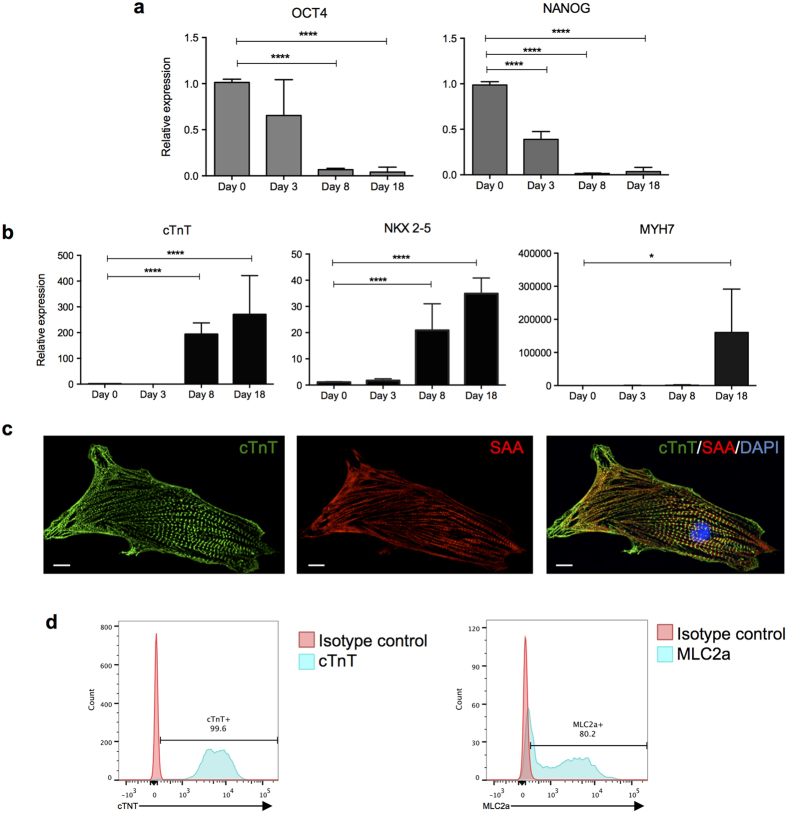
Characterization of hPSC-derived cardiomyocytes. (**a,b**) Relative gene expression of pluripotency (**a**) and cardiac (**b**) markers. The mean Ct values of duplicate measurements were normalized against the values for β actin for the same sample. After normalization, the means of three independent experiments were plotted, data represented are the mean ± SD. (**c**) Immunostaining of hPSC-derived CMs with cTnT and SAA antibodies followed by counterstaining with DAPI. (**d**) Flow cytometry analysis of cTnT and MLC2a expression. cTnT = cardiac Troponin T; MYH7 = myosin heavy chain beta; SAA = sarcomeric actinin alpha; MLC2a = myosin light chain 2a. *p < 0.05; ****p < 0.0001. Scale bar: 10 μm.

hPSC-CMs were generated from two independent cell lines (hES3 and NKX2-5^eGFP/w^) using inhibitors of GSK3 and Wnt in insulin-free medium^20^. Approximately 14 days after induction, spontaneously contracting cells were observed. RT-qPCR analysis revealed significant down-regulation of the pluripotency markers, hOCT-4 and hNANOG, from day 3 to day 8, with corresponding upregulation of the cardiac markers cTnT, NKX2-5 and MYH7, relative to undifferentiated hPSCs (Fig. 1a,b and Supplementary Fig. 1). Immunostaining at day 18 demonstrated that hPSC-CMs expressed cTnT and sarcomeric-α actinin (Fig. 1c and Supplementary Fig. 1). Flow cytometry analysis indicated that the hPSC-CMs were highly pure cellular populations, with more than 80% of cells expressing the cardiac atrial and ventricular marker MLC2a, and more than 98% of cells expressing cTnT (Fig. 1d and Supplementary Fig. 1). More than 70% of cells expressed the cardiac ventricular marker MLC2v, suggesting that the CMs were predominantly ventricular-like (Supplementary Fig. 2).

### Doxorubicin triggers cell death with apoptotic and necrotic features in hPSC-derived CMs

**Figure 2:**
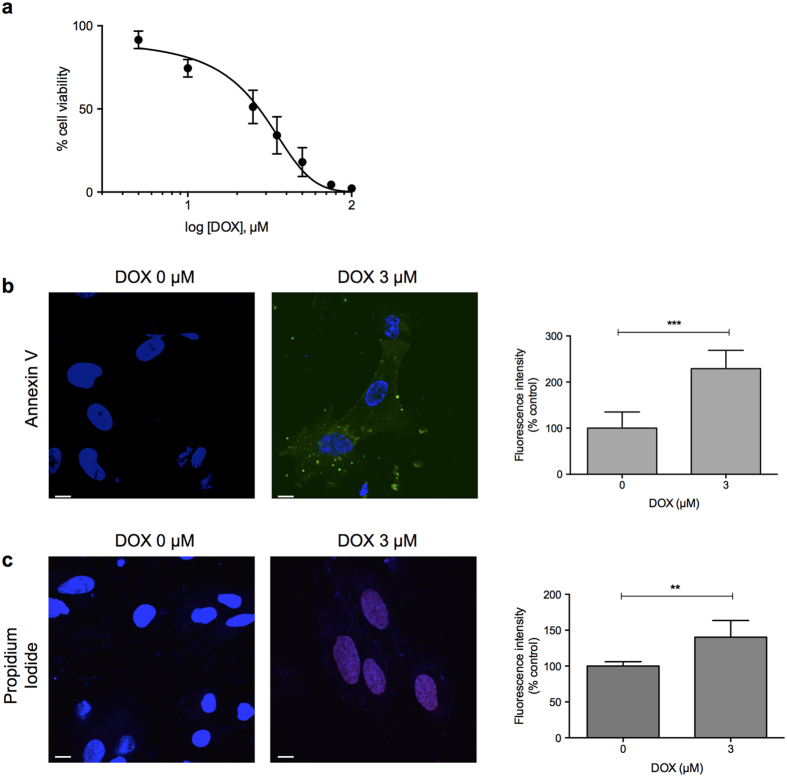
Characterization of doxorubicin-induced cell death in hPSC-derived cardiomyocytes. (**a**) Cell viability of hPSC-derived cardiomyocytes was assessed by measurement of ATP levels using CellTiter-Glo^®^ after 24 h of doxorubicin treatment. (**b,c**) hPSC-derived cardiomyocytes were treated with 3 μM of doxorubicin for 16 h and stained with Annexin V (**b**) or Propidium Iodide (**c**). Cells were counterstained with DAPI and analyzed by confocal microscopy. The total numbers of Annexin V and PI-positive cells as a percentage of control are shown in the bar graphs representing the means of three independent experiments ± SD. **p < 0.01; ***p < 0.001. Scale bar: 10 μm.

In humans, doxorubicin causes cell death of CMs within hours of administration^25^. We investigated doxorubicin-induced cytotoxicity in hPSC-CMs using assays of apoptotic and necrotic cell death. We observed that exposure of CMs to doxorubicin for 24 hours resulted in a dose-dependent decrease in cell viability compared to vehicle-treated control cells with an IC_50_ of 30.1 μM (Fig. 2a). Notably, cell viability began to decrease at doses as low as 5 μM, which overlaps with the peak concentration of the drug observed in plasma of patients after intravenous administration^26^, suggesting that this represents a clinically-relevant effect. We assessed cell death pathways using Annexin V (early apoptosis) and propidium iodide (late apoptosis/necrosis) staining. As shown in Fig. 2b,c, compared to vehicle treated cells, CMs treated with 3 μM of doxorubicin displayed increased staining of both Annexin V (229% of vehicle control, p-value 0.0005) and propidium iodide; (140% of vehicle control, p-value 0.003).

### Doxorubicin leads to mitochondrial dysfunction and ROS production in hPSC-derived CMs

**Figure 3:**
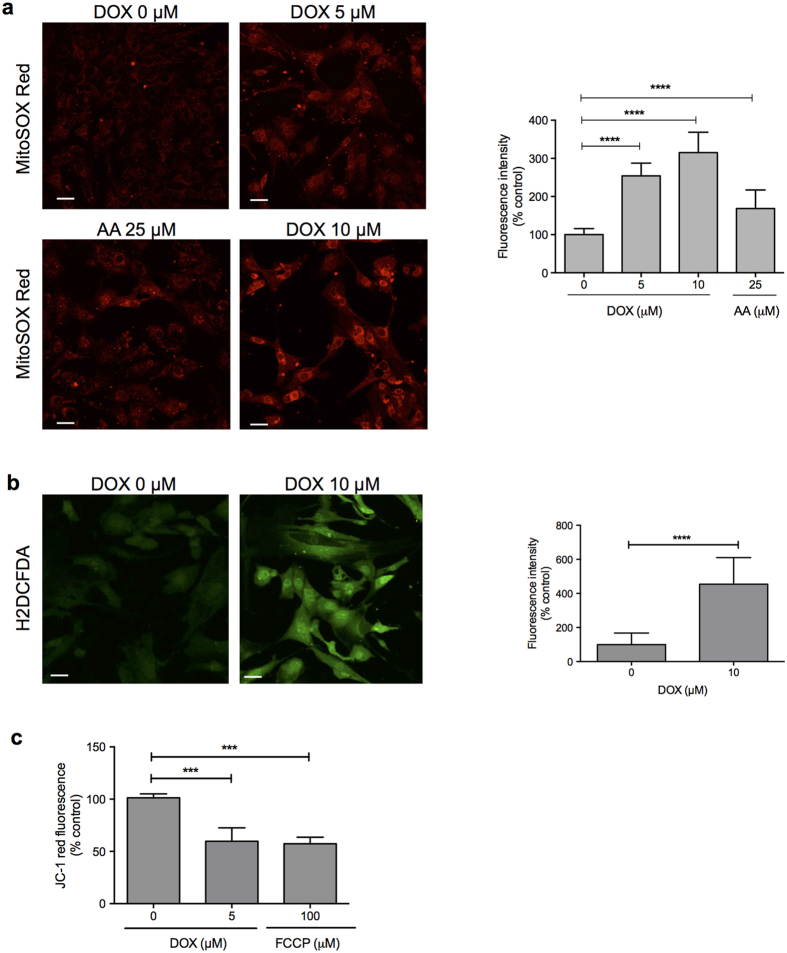
Doxorubicin triggers mitochondrial perturbations and reactive oxygen species (ROS) production in hPSC-derived cardiomyocytes. (**a**) Cells were incubated with doxorubicin or antimycin A (positive control) for 1 h and intra-mitochondrial O_2_^−^ production was detected using the fluorescent dye MitoSOX^TM^ Red. The total numbers of MitoSOX^TM^ Red-positive cells as a percentage of control is shown in the bar graph. (**b**) After 4 h of doxorubicin treatment, cells were washed and incubated with H2DCFHDA for intracellular H_2_O_2_ detection by confocal microscopy. The total numbers of H2DCFHDA-positive cells as a percentage of control is shown in the bar graph. (**c**) Doxorubicin and FCCP (positive control) treated cells were incubated with the potential-sensitive probe JC-1 and ΔΨm was analyzed in a plate reader with excitation set at 535 nm and emission at 590 nm. Graphs represent the means of three independent experiments ± SD. AA = antimycin A; FCCP = carbonyl cyanide *p*-trifluoromethoxyphenylhydrazone. ***p < 0.001; ****p < 0.0001. Scale bar: 30 μm.

ROS generation and mitochondrial disturbance are thought to be important processes in the pathogenesis of DIC^27^,^28^. We assessed the production of intracellular ROS species after doxorubicin treatment using two different fluorescent probes (MitoSOX Red and H2DCFDA). Antimycin A was used as a positive control for mitochondrial ROS production. Doxorubicin treatment led to an increase in intra-mitochondrial O_2_^−^ levels in a dose-dependent manner in hPSC-derived CMs (Fig. 3a). Furthermore, exposure of hPSC-derived CMs to doxorubicin also resulted in a significant increase in H_2_O_2_ production as measured by the H2DCFDA probe (Fig. 3b). To further examine mitochondrial function, we assessed the transmembrane potential (ΔΨm). ΔΨm is critical for maintaining the physiological function of the respiratory chain to generate ATP; loss of ΔΨm renders cells depleted of energy and can lead to subsequent cell death^29^. We observed a significant decrease in ΔΨm after 1 h of treatment with 5 μM of doxorubicin (Fig. 3c), to a similar extent as the positive control probe FCCP (Carbonyl cyanide-*p*-trifluoromethoxyphenylhydrazone). These data indicate that doxorubicin treatment causes acute impairments in mitochondrial function in human CMs.

### Doxorubicin induces an increase in intracellular Ca^2+^ and the formation of double-strand DNA breaks

**Figure 4:**
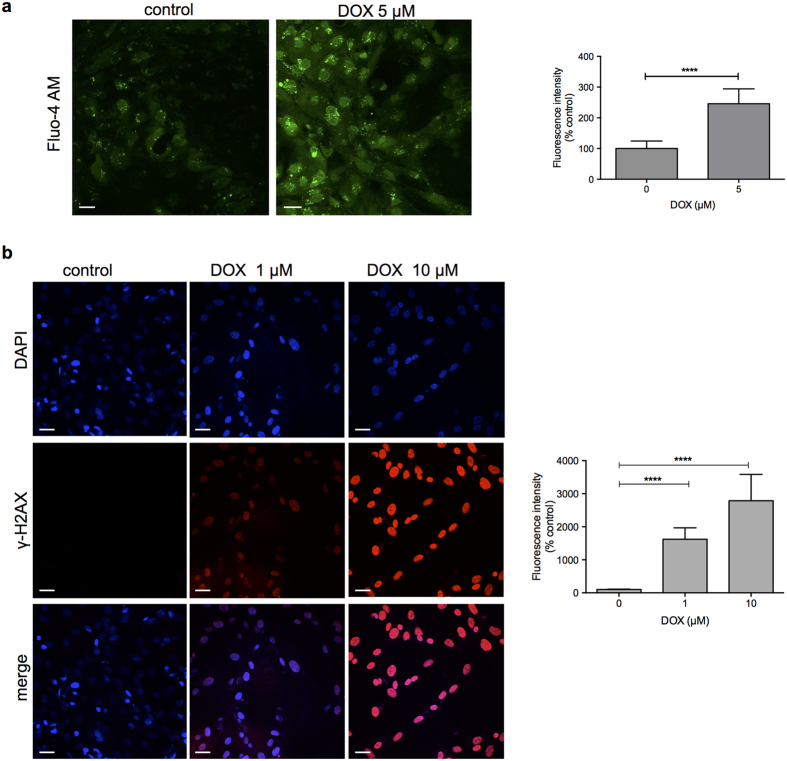
Doxorubicin induces intracellular Ca^2 + ^increase and double-strand DNA breaks (DSBs). (**a**) hPSC-derived cardiomyocytes were incubated with 5 μM of doxorubicin for 16 h and intracellular Ca^2+^ was measured using the cell-permeant fluorescent indicator Fluo-4, AM. The total numbers of Fluo-4, AM-positive cells as a percentage of control is shown in the bar graph. (**b**) Representative images of DSBs in hPSC-derived cardiomyocytes. γ-H2AX staining is shown in red, and DAPI nuclear staining in blue. γ-H2AX fluorescence intensity as a percentage of control is shown in the bar graph. ****p < 0.0001. Scale bar: 30 μm.

Doxorubicin-mediated alteration of Ca^2+^ homeostasis has been proposed as a mechanism of cardiotoxicity^4^,^30^. Intracellular Ca^2+^ accumulation is thought to initiate myocardial injury and impair contractile function^31^. We investigated intracellular Ca^2+^ concentration ([Ca^2+^]i) in hPSC-derived CMs after doxorubicin treatment using the Fluo-4, AM probe. As shown in Fig. 4a, [Ca^2+^]i level before treatment was relatively low and increased 2.5 fold after treatment with 5 μM of doxorubicin for 16 h. We next investigated the activation of DNA damage response pathway by doxorubicin in hPSC-derived CMs. A very early and sensitive marker of DNA double-stranded break (DSB) induction is the phosphorylation of histone H2AX on serine 139 (γ-H2AX). As shown in Fig. 4b, doxorubicin treatment induced the DNA damage signal γ-H2AX in hPSC-derived CMs in a dose-dependent manner.

### Multi-electrode array electrophysiology and response to doxorubicin

**Figure 5:**
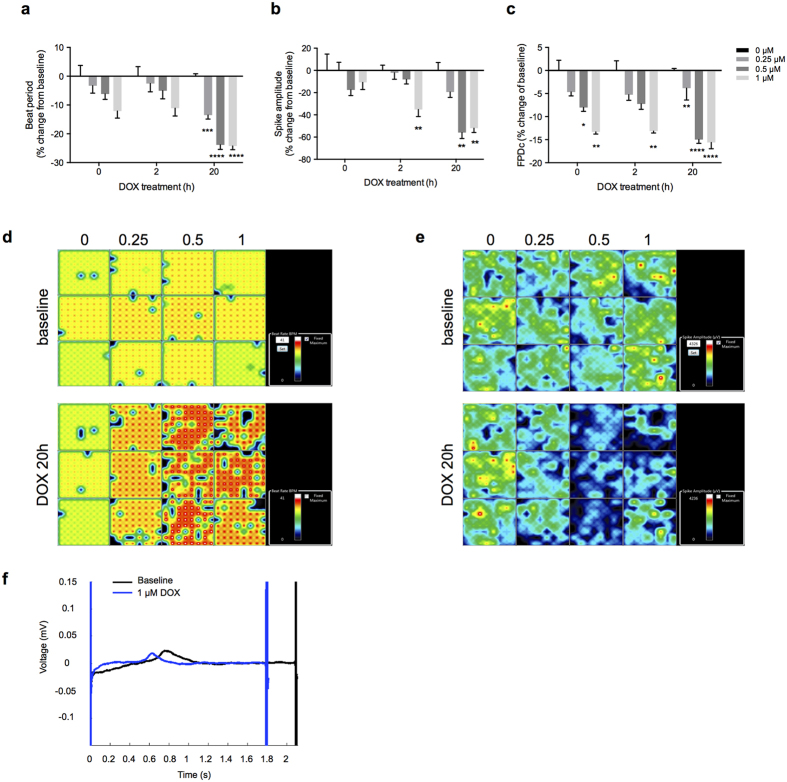
Doxorubicin alters the electrophysiological properties of hPSC-derived cardiomyocytes. (**a**–**c**) iCell^®^ cardiomyocytes were treated with doxorubicin in the indicated doses, and assessed by multi-electrode array technology immediately after treatment, and 2 h and 20 h after treatment. Doxorubicin treatment resulted in dose-dependent effects on beat period (**a**), spike amplitude (**b**), and FPDc (**c**). Graphs represent the means of three biological replicates ± SEM. (**d**) Activity maps displaying the beat rate at baseline and 20 h post-DOX treatment. (**e**) Activity map showing the spike amplitude at baseline and 20 h post-DOX treatment. (**f**) Beat waveform trace overlay at baseline and after 2 h of 1 μM DOX treatment. FPDc = corrected field potential duration. *p < 0.05; **p < 0.01; ***p < 0.001, ****p < 0.0001.

Doxorubicin has been associated with a number of different arrhythmias in humans, including sinus tachycardia, atrial fibrillation, T wave flattening, abnormalities of the QT interval, and atrio-ventricular nodal block^32^,^33^,^34^,^35^,^36^. To gain insight into electrophysiological effects of this drug, we performed multi-electrode array (MEA) analysis of hPSC-derived CMs before and after doxorubicin treatment. Immediately after treatment with doxorubicin, we observed a dose-dependent decrease in beat period and spike amplitude (Fig. 5a,b). These effects were seen at doses as low as 0.25 μM of doxorubicin, and the effects increased further with time. By 20 hours of doxorubicin treatment, beat period had decreased by 13.5% (DOX 0.25 μM) and 24.2% (DOX 1 μM) compared to baseline (Fig. 5a). Similarly, spike amplitude decreased by 19.4% (DOX 0.25 μM) and 52.1% (DOX 1 μM) compared to baseline (Fig. 5b). Activity maps of beat rate (Fig. 5d) and spike amplitude (Fig. 5e) were consistent with the dose- and time-dependent effects of doxorubicin on these parameters. Furthermore, doxorubicin treatment resulted in a dose-dependent reduction in the corrected field potential duration (cFPD), a correlate of the QTc interval on a surface electrocardiogram (Fig. 5c). The cFPD was reduced immediately after treatment with doxorubicin in a dose-dependent manner, and this reduction was stable throughout 20 hours of treatment (Fig. 5c). Analysis of the beat waveform confirmed the earlier repolarization caused by doxorubicin treatment (Fig. 5f). Collectively, these data establish that doxorubicin has both acute and chronic effects on electrophysiological parameters in hPSC-derived CMs, including reductions in beat rate, spike amplitude and cFPD.

### Effects of doxorubicin on gene expression

**Figure 6:**
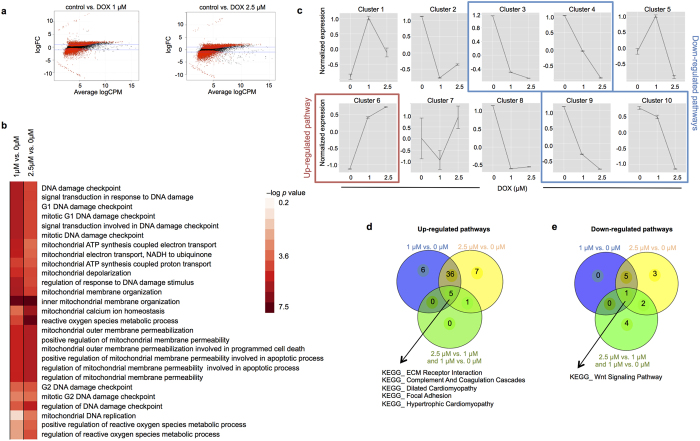
Whole genome transcript profiling identifies doxorubicin-dependent changes in gene expression in human cardiomyocytes. (**a**) Mean-average plots displaying the number of differentially expressed genes in hPSC-derived cardiomyocytes at indicated concentrations vs. control (analysis in edgeR). (**b**) Focused global test analysis of ROS, DNA damage and mitochondrial pathways summarizing the pathways significance in the groups 1 μM vs. 0 μM and 2.5 μM vs. 0 μM. (**c**) Spatially ordered clustering of gene expression profiles across increasing doxorubicin concentrations as determined by self-organizing maps (SOM). The clusters highlighted in red and blue were selected for pathway over-representation analysis via DAVID. (**d,e**) Comparative analysis of the number of significantly up- (**d**) and down (**e**) -regulated KEGG pathways (FDR < 0.05) determined either by PreRanked GSEA (1 μM drug vs. control and 2.5 μM drug vs. control) or via DAVID (dose-dependent changes, SOM cluster 6 for up-regulated genes and clusters 3, 4 and 9 for down-regulated genes). Number of pathways up- and down-regulated by doxorubicin were determined by PreRankedGSEA analysis.

To gain additional insight into the molecular mechanisms and pathways involved in DIC, we performed whole genome transcriptome profiling via RNA-seq in hPSC-derived CMs treated with doxorubicin or vehicle only control. Cells were treated with 0, 1 or 2.5 μM of doxorubicin for 16 hours and RNA was isolated for high throughput sequencing. These doses were chosen because they were not expected to result in significant cell death. To identify genes that are significantly regulated by doxorubicin treatment, we analyzed read count data from RNA-seq using edgeR software. In comparison to control treated cells, treatment with 1 μM or 2.5 μM of doxorubicin resulted in up-regulation of 2,767 and 2,939 transcripts, respectively, and down-regulation of 3,793 and 4,038 transcripts, respectively, at a false discovery rate (FDR) <0.05 (Fig. 6a). We performed KEGG pathway analysis using PreRankedGSEA and identified 41 pathways that were up-regulated by both treatment conditions (Supplemental Table 1), and 6 pathways that were down-regulated by both treatment conditions (Supplemental Table 2). Intriguingly, several of the up-regulated pathways were enriched for a common set of structural genes related to various cardiomyopathies, and included genes from the integrin, troponin and tropomyosin families (Supplementary Table 1). A GO analysis focused on genes involved in ROS production, DNA damage and mitochondrial pathways showed strong evidence of upregulation of these pathways by doxorubicin (Fig. 6b).

We next examined dose-dependent changes in gene expression by conducting an ANOVA with post-hoc contrasts in edgeR and identified transcripts that displayed significantly different expression between both the 1 μM vs. control and 2.5 μM vs. 1 μM treatment conditions (FDR < 0.05). Under this condition, no genes showed evidence for statistically significant dose-dependent upregulation in response to doxorubicin. In contrast, 210 genes showed evidence of dose-dependent down regulation in response to doxorubicin. We then used self-organized maps to identify patterns of gene expression across the three treatment groups (Fig. 6c). Four SOM clusters (clusters 3, 4, 9 and 10) showed evidence of coordinate down-regulation in a dose-dependent manner and almost entirely overlapped with the 210 genes found to be significant in ANOVA. Pathway analysis on these 210 genes demonstrated over-representation of pathways related to cell growth and proliferation, including Wnt signaling, TGF-beta signaling, and various forms of cancer. One gene cluster (cluster 6) showed evidence of a near-monotonic dose-dependent increase in gene expression. Pathway enrichment analysis on this gene cluster confirmed the presence of gene-sets related to structural organization in cells.

Finally, we compared the significantly enriched up- and down-regulated pathways (FDR < 0.05) obtained when examining the effects of both drug concentrations against control treatment (PreRankedGSEA analysis) or when restricting the analysis only to genes showing dose-dependent changes in expression (DAVID analysis). This analysis highlighted the structural gene-based cardiomyopathy pathways as being commonly upregulated among the three conditions (Fig. 6d). Conversely, the Wnt signaling pathway was found to be down-regulated in all conditions (Fig. 6e).

### *TOP2B* is essential for doxorubicin-induced cell death in hPSC-derived cardiomyocytes

**Figure 7:**
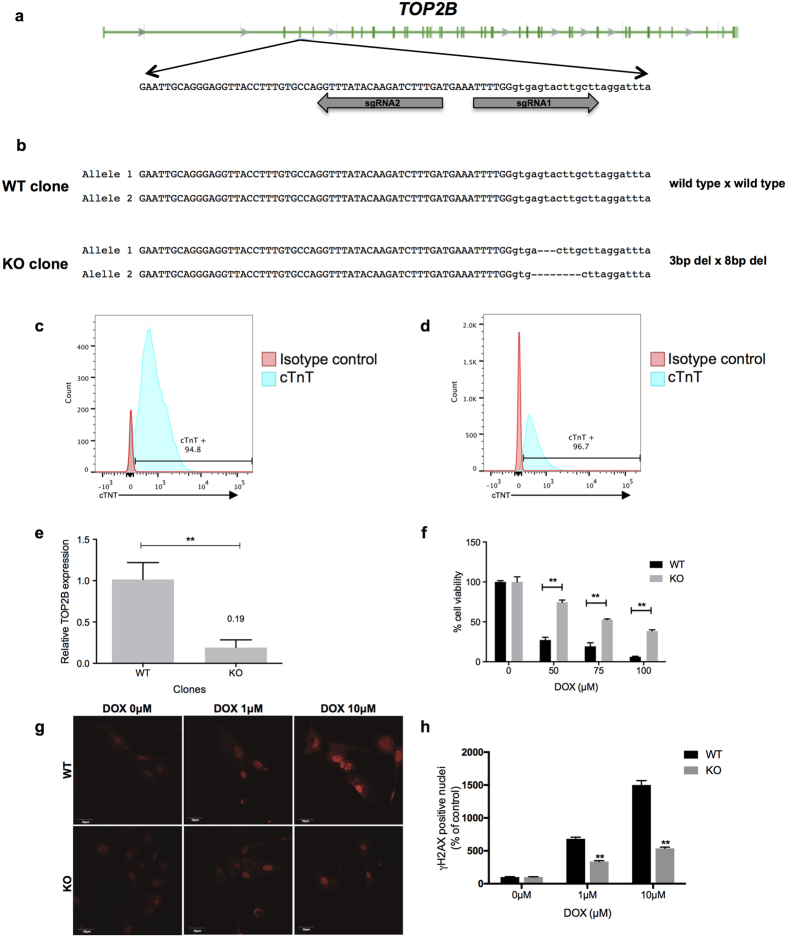
Disruption of *TOP2B* in hPSC-derived cardiomyocytes decreases sensitivity to doxorubicin-induced cell death. (**a**) Schematic of the *TOP2B* gene showing location of single guide RNAs (sgRNAs) designed to recognize the 3′ region of exon 3. Exonic sequence is shown in uppercase and intronic sequence in lowercase. (**b**) Sequences of two clones transfected with CRISPR-Cas9 targeting *TOP2B* gene showing either wild type sequence (WT), or bi-allelic deletions resulting in gene knock-out (KO). (**c,d**) Flow cytometry analysis of cTnT expression in wild type (**c**) and KO (**d**) clones. (**e**) Relative gene expression levels of *TOP2B* mRNA in the genome edited-cardiomyocytes. Data represent the mean Ct values of duplicate measurements normalized against the values obtained for β actin for the same sample. (**f**) After cardiomyocyte differentiation, cell viability of *TOP2B* edited-clones treated with doxorubicin for 24 hours was measured using CellTiter-Glo^®^ luminescent assay. (**g**) Wild type and knock out TOP2B cardiomyocytes were treated with doxorubicin and stained with γ-H2AX to identify double strand DNA breaks. (**h**) Quantification of data in panel (**g**). Graphs represent the means of three independent experiments ± SD **p < 0.01 Scale bar: 10 μ m.

*Top2B* has been shown to be essential for doxorubicin-induced cell death in mouse cells and rat CMs^37^. In addition, a mouse model with cardiac-specific deletion of *Top2b* was protected from DIC^5^. However, no previous study has examined the role of *TOP2B* in DIC in human CMs. We performed genome editing in hPSCs via CRISPR-Cas9 to inactivate the *TOP2B* gene. We choose to examine *TOP2B* because of the substantial data implicating this gene in the pathogenesis of DIC in murine systems^5^,^37^, which suggested that it may also play a role in DIC in human CMs. Using a guide RNA (gRNA) construct targeting exon 3 of the *TOP2B* gene (Fig. 7a), we generated a clonal population of cells with bi-allelic splice site mutations in exon 3 of the *TOP2B* gene, predicted to lead to premature truncation of the encoded protein (Fig. 7b). Sequencing of predicted off-target sites elsewhere in the genome did not reveal evidence of any off-target activity of the gRNA construct (Supplementary Fig. 3). Flow cytometry analysis showed that both wild-type and knock-out (KO) clones expressed high level of cTnT after CM differentiation (94.8% and 96.7% respectively, Fig. 7c,d). RT-PCR analysis confirmed that *TOP2B* RNA expression was reduced by >80% relative to wild-type hPSC-CMs (Fig. 7e). Following directed differentiation of wild-type and genome edited hPSCs to CMs, we treated them with doxorubicin for 24 hours and assessed cell viability. CMs with disruption of *TOP2B* displayed significantly decreased susceptibility to doxorubicin-induced cell death (Fig. 7f). In addition, CMs in which *TOP2B* was disrupted had substantially abrogated γ-H2AX staining after doxorubicin treatment (Fig. 7g,h), indicating that these cells were resistant to doxorubicin-induced DSBs. These results establish a *TOP2B*-dependent mechanism of doxorubicin-induced DSBs and cell death in human CMs.

## Discussion

Here we investigated the features of DIC using a novel human hPSC-derived CM model. We demonstrated that doxorubicin treatment led to a decrease in cell viability with apoptotic and necrotic characteristics, in agreement with previous reports from cellular and human biopsy studies^5^,^38^,^39^. Within hours of doxorubicin treatment, we observed significant production of ROS, mitochondrial dysfunction and increased [Ca^2+^]i in our hPSC-derived CMs, mechanisms that have been previously described as features of early onset doxorubicin-induced cell death^27^,^28^,^40^. This model therefore recapitulates many of the known cardinal features of DIC in a human cellular system.

Doxorubicin is known to cause various different arrhythmias in humans as well as rat CMs^35^,^41^,^42^. Previous studies have investigated the effects of doxorubicin and other anthracyclines on arrhythmogenicity in mouse embryonic stem cell-derived CMs and induced pluripotent stem cells-derived cardiomyocytes, and reported that these drugs resulted in reduced beating rate and abnormal beating pattern^42^,^43^,^44^. Using MEA technology, we showed that doxorubicin significantly altered the electrophysiological properties of hPSC-derived CMs with time- and dose-dependent decrease in spike amplitude, increase in beat rate and shortening of cFPD. Notably, these effects were observed immediately after exposure to doxorubicin, and worsened with prolonged treatment. Our results indicate that doxorubicin has both acute and chronic effects on electrophysiological parameters in human CMs. Our data are in agreement with a recent report showing that doxorubicin causes a decrease in beat period, spike amplitude and cFPD in a dose-dependent manner^45^. In addition, our data establish the time-dependency of these effects induced by doxorubicin treatment for the first time. It is notable that the cardiotoxic effects of doxorubicin could be detected by MEA at doses as low as 0.25 μM and appeared immediately after treatment with the drug. This finding re-inforces the utility of MEA technology as a highly sensitive tool to study drug toxicity.

We performed transcriptome profiling to identify specific molecular pathways affected by doxorubicin treatment. Interestingly, we found that doxorubicin treatment caused up regulation of structural genes associated with several cardiomyopathy-related pathways, including dilated and hypertrophic cardiomyopathy. This suggests that doxorubicin induces a broad program of gene expression associated with cellular remodeling and impaired cardiomyocyte function, and suggests that DIC may share common pathogenic mechanisms with these other forms of cardiomyopathy. DIC is known to share pathological and functional similarities with dilated cardiomyopathy^46^, and our results showing upregulation of genes in the dilated cardiomyopathy pathway induced by doxorubicin provides a mechanistic basis for that observation. By performing a GO analysis, we demonstrated that doxorubicin induced an up-regulation of genes involved in ROS production, DNA damage and mitochondrial pathways, which is in agreement with the data obtained in our cellular assays. These results are consistent with a recent study that similarly showed up-regulated GO pathways enriched in DNA damage stimulus and oxidative stress response^47^. However, in that study the authors reported doxorubicin led to down-regulation of cardiomyopathy-related pathways, in contrast to our finding that doxorubicin increased these gene pathways. The reasons for this discrepancy are unclear, but may relate to the lower doses of the drug and longer exposure in that study^47^, reflecting a difference between the acute versus chronic effects of the drug. Our clustering analysis also showed evidence of coordinate down-regulation of pathways related to cell growth and cancer. This is in agreement with results of microarray analysis that reported down-regulation of gene expression in pathways related to cancer and cellular senescence after doxorubicin treatment of human cardiomyocytes^48^.

The anticancer effects of doxorubicin are believed to occur through the inhibition of topoisomerase-II enzymes (Top2β and Top2α) and the subsequent blockage of DNA resealing during replication that eventually leads to cell death of cancer cells^49^. After entering the cells, doxorubicin binds to its cellular target topoisomerase-II and forms a doxorubicin-Top2-DNA ternary cleavage complex^49^. This complex can induce DSBs in DNA and eventually leads to cell death^5^,^50^. CMs express *TOP2B* but not *TOP2A*, and recently, *Top2B* was shown to be critical for DIC in a mouse model^5^. However, the role of *TOP2B* in response to doxorubicin in human CMs has not been previously studied. Using CRISPR-Cas9, we showed that disruption of *TOP2B* reduces the sensitivity of hPSC-CMs to doxorubicin-induced DSBs and cell death. These data establish a critical role of *TOP2B* in the pathogenesis of DIC in human CMs. These results also point to the utility of genome editing in hPSC-CMs as a method to assess the function of specific genetic variants. This approach may be useful to investigate the functional consequences of genetic variants that have been associated with DIC from human genetic association studies^51^,^52^.

Despite their evident advantages, hPSC-derived CMs have several limitations that merit consideration. Firstly, hPSC-derived CMs are less mature than native adult myocytes and are most analogous to a fetal stage of development^53^. Secondly, common hPSC differentiation protocols create a mixture of cells atrial-, ventricular- and nodal-like phenotypes^54^. Finally, hPSC-derived CMs are single-cell models and lack three dimensional tissue interactions of native myocardium. Advances in differentiation protocols, including maturation techniques and 3D cultures are likely to overcome some of these limitations^55^,^56^,^57^,^58^,^59^. As these techniques advance, there will likely be increased utility of hPSC-derived CMs in human disease modeling, high throughput screening, drug discovery and predictive cardiotoxicology. An additional limitation of our study is that we did not determine the specificity of doxorubicin in leading to the observed cardiotoxicity. Future studies could use the hPSC-derived CM model system to compare doxorubicin to other cardiotoxic drugs to gain insight into this question.

In summary, our results establish the characteristics of DIC in hPSC-derived cardiomyocytes. This model recapitulates many of the salient features of DIC observed in humans, suggesting that it may allow physiologically accurate dissection of the molecular mechanisms of DIC. This model is amenable to high-throughput applications, and could therefore enable screening of novel cardioprotective agents to mitigate the cardiotoxicity of doxorubicin. Together with genome-editing technologies, this model will also allow investigation of the functional impact of genetic variants on doxorubicin-response. Finally, by using genome-edited or patient-specific pluripotent stem cells, this strategy could allow personalized approaches to predicting the risk of toxicity in individual patients, prior to the administration of doxorubicin.

## Methods

### Cell culture and cardiomyocyte differentiation

HES3 cell lines were obtained under license from the WiCell Research Institute and NKX2-5^eGFP/w^ hESCs were a generous gift from Dr. David Elliott^60^. Undifferentiated hESCs were grown in mTeSR™1 (Stemcell Technologies) on Matrigel-coated plates and passaged every 4 days using 0.02% EDTA. Cells were passaged for at least five times before beginning differentiation to obtain a monolayer of cells. hPSC were differentiated into cardiomyocytes according to the protocol described by Lian *et al.*^20^. Briefly, cells were seeded on matrigel-coated 12-well plates and 4 days later incubated in RPMI/B27 (Life Technologies) without insulin + CHIR99021 (Selleckchem) (10 or 12 μM). After 2 days, medium was changed to RPMI/B27 without insulin with 5 μM of IWP2 (Tocris). 48 h later, medium was changed to fresh RPMI/B27 without insulin and RPMI/B27 with insulin was added 2 days later. Beating clusters were observed after 7 to 14 days. For electrophysiological studies using the Axion MEA, iCell™ cardiomyocytes were obtained from Cellular Dynamics International and recovered from frozen culture as recommended by the manufacturer. iCell cardiomyocytes were allowed to recover for 12 days in iCell Maintenance Medium (Cellular Dynamics International) before experimentation.

### Quantitative realtime PCR

Following RNA extraction (RNeasy mini kit, Qiagen) and cDNA synthesis (Superscript^®^ II Reverse Transcription Kit, Life Technologies), quantitative realtime PCR was carried out on the StepOnePlus™ (Applied Biosystem) using SYBR^®^ Select Master Mix (Life Technologies). Each sample was run in triplicate and each reaction contained 5 ng of cDNA in a total volume of 20 μL. The concentration of mRNA was normalized to the geometric mean of mRNA of β actin, the reference gene, and fold change was calculated using the ΔΔC_t_ method. Primer sequences are shown in Supplementary Table 3.

### Immunostaining

hPSC-derived cardiomyocytes were seeded on Matrigel-coated 20 mm coverslips in 12-well plates. Cells were washed with 1× PBS and fixed with 4% formaldehyde in PBS at room temperature for 10 min. The coverslips were washed and blocked with blocking buffer (1× PBS + 0.3% TritonX + 3% goat serum) at room temperature for 30 min. For staining, the following antibodies and concentration were used: anti-cTnT (ab10214, Abcam) 1:400, anti-SAA (ab9465, Abcam) 1:200, anti-Mlc2a (311 011, Synaptic Systems) 1:200 or anti-γ H2AX phosphoS139 (ab2893, Abcam). Antibodies were diluted in 1× PBS + 0.3% TritonX + 2% goat serum and incubated at 4 °C overnight. The cells were then washed three times for 5 min and stained with secondary antibodies (Alexa Fluor^®^ 488 or 647, Invitrogen) at a dilution of 1:200. The cells were stained with DAPI for 10 min in 1× PBS and after two more washes mounted onto microscope slides using ProLong^®^ Gold Antifade Mountant (Life Technologies).

### Flow cytometry

hPSC-derived cardiomyocytes were trypsinized and fixed in 1% formaldehyde at room temperature for 20 min. Cells were washed and incubated in PBS + 1% FCS with anti-cTnT (ab45932, Abcam) and anti-Mlc2a (311011, Synaptic Systems) 1:200 overnight at 4 °C. Subsequently, cells were stained with secondary antibodies (Alexa Fluor^®^ 488 or 555, Invitrogen) 1:200 for 30 min on ice. After washing, cells were resuspended in 1× PBS + 1% FCS and analyzed on a BD FACSCanto™ II flow cytometer.

### Cell viability assays

Cells seeded on a Matrigel-coated 96-well culture plate were treated with increasing doses of doxorubicin for 24 h. After treatment, cells were washed once with 1× PBS and incubated with CellTiter^®^-Glo buffer (Promega) on a shaker at room temperature for 10 min. Cells were washed once with 1× PBS and incubated in fresh medium before luciferase signal was taken.

### ROS production and calcium handling

Cells were plated on Matrigel-coated 35 mm glass bottom dishes and treated with doxorubicin the following day. After treatment, cells were washed once with 1× PBS and incubated 15 min at 37 °C in plain RPMI with 5 mM MitoSOX™ Red, H2DCFDA or Fluo-4, AM. Antimycin A (25 μM; Sigma-Aldrich) was used as a positive control for mitochondrial ROS production. Cells were washed and analysed on an Olympus FV1000 inverted confocal microscope.

### Mitochondrial transmembrane potential

The mitochondrial transmembrane potential (Δψm)-sensitive probe JC-1 (ab113850, Abcam) was following manufacturer’s instructions. Briefly, cells were incubated with JC-1 (1 μM) for 10 min at 37 °C. As a positive control, cells were incubated separately with 100 μM of FCCP, an uncoupling agent. Cells were analyzed in a plate reader, with excitation set at 535 nm or 475 nm and emission at 590 nm.

### Propidium iodide and Annexin V staining

After treatment with doxorubicin, cells were washed with 1× PBS and incubated in 100 μL of Annexin V buffer. 5 μL of Annexin V Alexa Fluor 488 (A13201, Molecular Probes) was added and cells incubated in the dark for 15 min at room temperature. Alternatively, cells were incubated with 2 μg/mL of propidium iodide in Annexin V buffer. Cells were washed and stained with DAPI before analysis.

### Genome editing

A pair of single guide RNAs (sgRNA#1:TTTTGGGTGAGTACTTGCTT and sgRNA#2: TCAAAGATCTTGTATAAACC) targeting exon 3 of the *TOP2B* geme were cloned into the BbsI site of pSpCas9n(BB)-2A-Puro (PX462) (Addgene). 2.5 μg of plasmid DNA was introduced into 1 × 10^6^ NKX2-5^eGFP/w^ cells by electroporation (1600 volts, 10 ms pulse width, 2 pulses) using a Neon^®^ Transfection System (Life Technologies). After electroporation the cells were plated on a Matrigel-coated 6 well plate and after 24 h treated with 1 μg/mL puromycin. Twenty-four hours later the antibiotic was removed and isolated colonies were picked manually approximately 2 weeks later. Genomic DNA was extracted and 200 ng was used for PCR amplification of the *TOP2B* gene. Each 50 μL PCR reaction contained 5 μL of 2.5× Accuprime™ buffer, 400 nM of primer (sequences available on request), 0.4 U of Accuprime™ Taq DNA polymerase (Life Technologies) and nuclease-free water. To detect mutations, we performed the Surveyor nuclease assay using 25 μL of PCR product with 1 μL of Surveyor nuclease, 1 μL of Surveyor Enhancer, and 3 μL of 0.15 M MgCl_2_ in a 50 μL reaction (Transgenomic SURVEYOR mutation detection kit for standard gel electrophoresis, Transgenomic^®^). Surveyor nuclease digestion was assessed by gel electrophoresis. The purified PCR products corresponding to the positive clones were cloned in pGem^®^-T easy vector (Promega) and transformed in One Shot^®^ TOP10 E.coli competent cells (Life Technologies). After ampicillin selection and amplification, DNA of at least 10 bacterial clones of each sample was extracted and subjected to Sanger sequencing.

### Multielectrode array recording and analysis

iCell Cardiomyocytes (Cellular Dynamics International) were thawed in Plating Medium and seeded onto a fibronectin-coated Maestro MEA 12-well plate (Axion Biosystems) at a density of 16,000 cells per well. Activity was recorded prior to treatment (baseline), just after drug treatment (0 h), and 2 h and 20 h post-treatment with doxorubicin using the Maestro MEA system (Axion Biosystems). DMSO was used as vehicle control and an equal volume of vehicle control or DOX was added to the wells. The recordings were taken at 37 °C using the standard cardiac settings (AxionBiosystems Maestro AxIS software version 2.1.1.5). Signals were filtered using a bandpass filter ranging from 0.1 Hz to 2 kHz, and the beat detection threshold was 300 uV. Field potentials were analyzed with the platform software and outputs included beat period (sec), spike amplitude (μV) and field potential duration (ms).

### RNA-Seq

Two sets of duplicate samples of hPSC-derived cardiomyocytes were treated with vehicle only, 1 μM or 2.5 μM of doxorubicin for 16 hours. Total RNA was extracted from control and doxorubicin-treated cardiomyocytes using TRizol reagent. RNA quality check and quantification was performed on an Agilent Technologies 2100 Bioanalyzer. All samples displayed a 260/280 ratio ≥1.8 and RNA integrity ≥9. One μg of RNA from each sample was used to generate cDNA libraries for RNA sequencing using the TruSeq RNA Sample Prep Kit v2 (Illumina). Sample preparation was performed according to the manufacturer’s protocol. Briefly, isolation of polyadenylated RNA molecules was performed using poly-T oligo attached magnetic beads, followed by enzymatic RNA fragmentation, cDNA synthesis, ligation of bar-coded adapters and PCR amplification. The amplified cDNA fragments were analyzed using the 2100 Bionalyzer to determine fragment quality and size. Six paired-end cDNA libraries were determined by the Sequencing library qPCR quantification from Illumina, normalized to 10 nM and sequenced on a Hiseq 2500 sequencer (Illumina).

Paired-end fastq sequence reads from each sample were assembled using the splice-aware read-mapper Tophat^61^. Aligned reads were mapped to the human reference genome (hg19 build) for transcript assembly and quantification using Cufflinks^62^. Differential expression of genes and transcripts between control and doxorubicin treated samples was determined using the edgeR software^63^. The transcriptome data from the 1 μM vs. control and 2.5 μM vs. control samples was investigated for common enrichment of biological pathways by querying ‘gene-sets’ from the Kyoto Encyclopedia of Genes and Genomes (KEGG)^64^ pathway repository via the PreRankedGSEA tool^65^. In a separate analysis, genes demonstrating dose-dependent changes in expression (1 μM vs. control and 2.5 μM vs. 1 μM) were identified via analysis of variance within edgeR and further analyzed for pathway over-representation via the Database for Annotation, Visualization and Integrated Discovery (DAVID) tool^66^. Significantly differentially expressed pathways were identified after controlling for the false discovery rate (FDR) at 5%. We employed self-organizing maps (SOM) to cluster and visualize the patterns of gene expression across the dosage ranges^67^. The focused analysis to identify pathways related to ROS, DNA damage and mitochondrial pathways was done using the R package ‘globaltest’ and Gene Ontology (GO).

### Statistical analysis

Fluorescent intensities were quantified using ImageJ software version 2.0.0. Data are expressed as means ± standard deviation (SD). Statistical significance was ascertained by one-way ANOVA with appropriate post hoc testing or by Student’s t test. Differences were considered statistically significant when p < 0.05.

## Additional Information

**How to cite this article**: Maillet, A. *et al.* Modeling Doxorubicin-Induced Cardiotoxicity in Human Pluripotent Stem Cell Derived-Cardiomyocytes. *Sci. Rep.* **6**, 25333; doi: 10.1038/srep25333 (2016).

## Supplementary Material

Supplementary Information
