# Supplementary material for: Modeling Doxorubicin-Induced Cardiotoxicity in Human Pluripotent Stem Cell Derived-Cardiomyocytes
Source: Sci Rep. 2016 May 4;6:25333. doi: 10.1038/srep25333 (PMC4855185; doi:10.1038/srep25333)
Supplement: Supplementary Information [file srep25333-s1.pdf]

MODELING DOXORUBICIN-INDUCED CARDIOTOXICITY IN HUMAN PLURIPOTENT STEM CELL DERIVED-CARDIOMYOCYTES

Authors:

Agnes Maillet, PhD, Kim Tan, BSc, Xiaoran Chai, PhD, Singh N. Sadananda, PhD, Ashish Mehta, PhD, Jolene Ooi, PhD, Michael R. Hayden, MD, PhD, FRCPC, Mahmoud A. Pouladi, MSc, PhD, Sujoy Ghosh, PhD, Winston Shim, PhD and Liam R. Brunham, MD, PhD, FRCPC

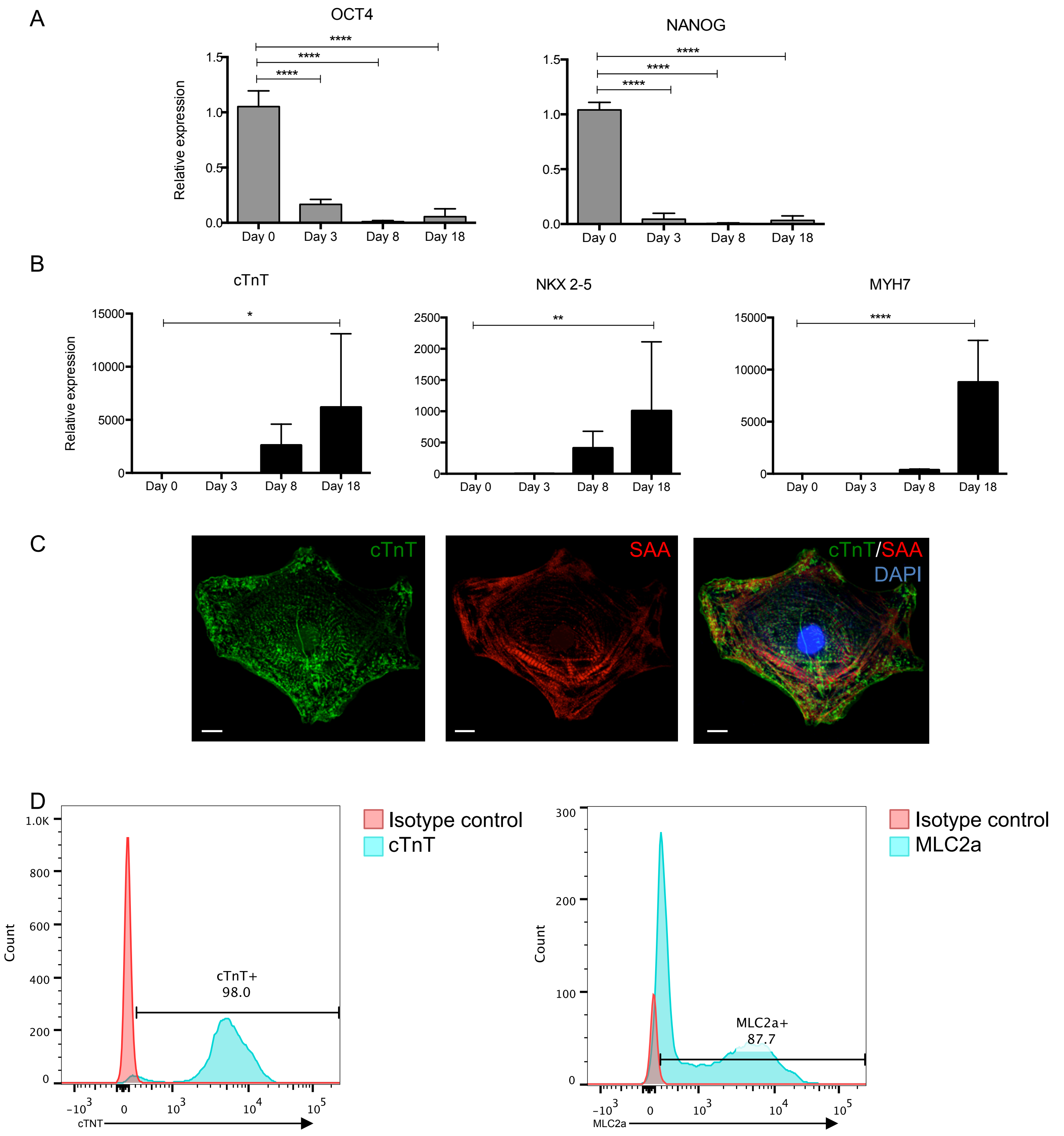

Supplementary figure 1: Characterization of hPSC-derived cardiomyocytes (NKX2-5$^{eGFP/w}$ cell line). A) Relative gene expression levels of pluripotency markers (A) and cardiac markers (B). The mean Ct values of duplicate measurements were normalized against the values obtained for β actin for the same sample. After normalization, the means of three independent experiments were plotted. Data represent the mean ± SD. C) Immunostaining of hPSC-derived CMs with cTnT and SAA antibodies followed by counterstaining with DAPI. D) Flow cytometry analysis of cTnT and MLC2a expression. cTnT: cardiac Troponin T, MYH7: myosin heavy chain beta; SAA: sarcomeric actinin alpha, MLC2a: myosin light chain 2a. *p<0.05; **p<0.01; ****p<0.0001. Scale bar: 10 μm

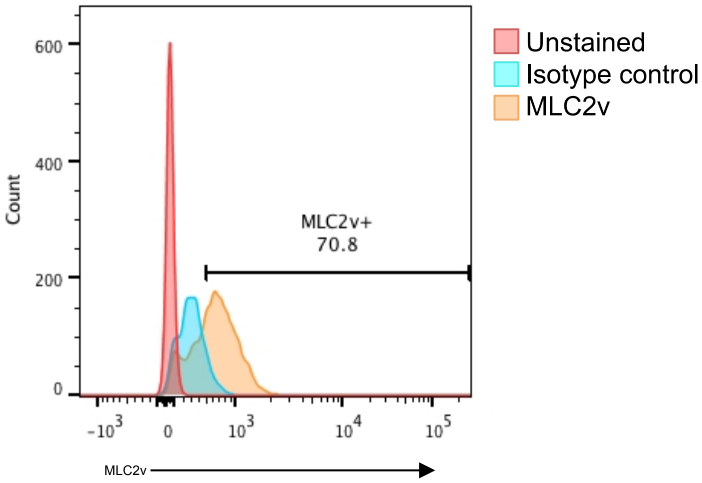

Supplementary figure 2: MLC2v expression in hPSC-derived cardiomyocytes (hES3 cell line). Flow cytometry analysis MLC2v expression revealed that a majority of the cardiomyocytes expressed the ventricular marker MLC2v, indicating that these cell are predominantly ventricular-like.

A

**A1**	TTCAGAGTTGAGGTTACCAGTCCATTTAAGGTAACATGGTATAGGTTGGGGGAGTACTTGCTTTAGGATA GTTGTGATGCTAAGTGTGTTAATGTCTATAGAATA	**Chr 7**<br>**Position 103002807**<br>**Score 2.603**
**A2**	TCCCAGTTGCCAAGGAAGGCCTGGGTCTTCTTAATTTGTTTTTAGTCCCAAAGCAAGTACTTACCTAAAC TGAATAGAGGCAGGAACTCTATCCAGTCTAAGCTCTC	**Chr 12**<br>**Position 56373518**<br>**Score 1.632**
**A3**	AATGGCTTTTCCTAAAATCCATTCATCAACCAGCAGGAAAAGACTAAAGCAAGTTCTCACCCGAGATTGG GAAGCAGAAAGTTAATGAGCTACCTGACATTCTCTCT	**Chr 2**<br>**Position 238524473**<br>**Score 1.384**
**A4**	TCCAATCCAAACACCTCATCTCTGAATTTTACAGCCTTTTCTGGGGGAGCACTTGCTTAGGAAAGAAAC ATTCAACCTTAAACCACCAGGGCAACGGGGACAAAGCT	**Chr 14**<br>**Position 77472439**<br>**Score 1.330**
**B1**	CAGTTAGACTATAGCCCTTGTGTATTTATAGTTTCCATGAATGTGTACTAAATGATTTTGTATAAACCAAGT ATTTGCATGTGAAACTTTTTCTGTCTCTAGTATCAT	**Chr 3**<br>**Position 197801471**<br>**Score 1.592**
**B2**	GAAGCACAAAATTCAGTAAGTCAAAAGCAAAGATTTCATCCCATGAGTCCAGGTTTATACATTTTCTTTGA CATGCAATGATTTTTAAGTAGTTTTATTCTTCATGTG	**Chr 20**<br>**Position 53396700**<br>**Score 1.305**
**B3**	TTTTGGGACTGGAAAATACAGAAGACTCCTTGGTTTATACATGGTCTTGTAAGAGAATCAGTACTTACAG CACTATTGCAATTCCAAGTATTGAAGCAATCGCAGGT	**Chr 6**<br>**Position 116560209**<br>**Score 1.278**
**B4**	GATCTTTATATTAAAGGATTTACTCACCTAGAATCTCATCAAAGATTTTGTACAAACCAGGAACAAAAGTG ACTTCCCTATAGTTAATGCCAACATCTTCATCGTAA	**Chr 17**<br>**Position 38572684**<br>**Score 1.095**

B

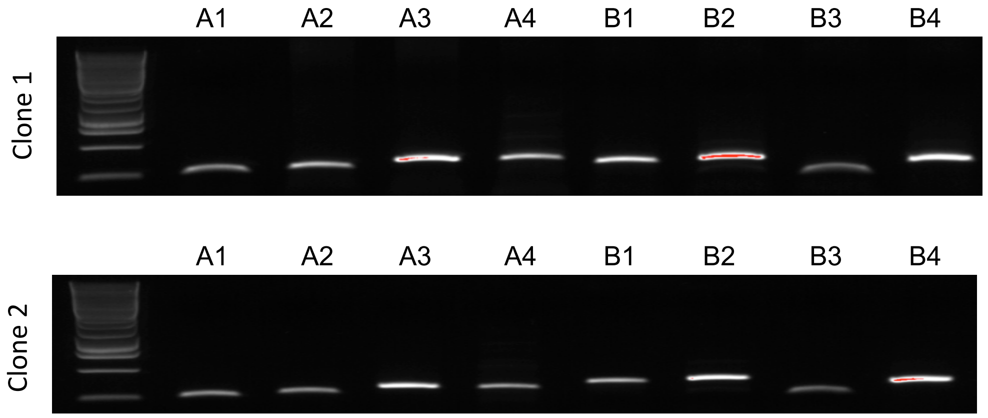

Supplementary figure 3: Analysis of the main potential off-target sites of Top2b CRISPR-Cas9. A) Potential off-target sites of Top2b-CRISPR gRNA1 (A1 to A4) and gRNA2 (B1 to B4). B) PCR amplification with primers specific to sequences A1 to B4. PCR products were purified and subjected to Sanger sequencing.

Supplementary Table 1: Pathways up-regulated by doxorubicin treatment

Pathway
KEGG_Ribosome
KEGG_ECM Receptor Interaction
KEGG_Lysosome
KEGG_Oxidative Phosphorylation
KEGG_Cell Adhesion Molecules CAM
KEGG_Alzheimers Disease
KEGG_Glutathione Metabolism
KEGG_Other Glycan Degradation
KEGG_Parkinsons Disease
KEGG_Cardiac Muscle Contraction
KEGG_Huntingtons Disease
KEGG_Complement And Coagulation Cascades
KEGG_Dilated Cardiomyopathy
KEGG_Valine Leucine And Isoleucine Degradation
KEGG_Focal Adhesion
KEGG_Arginine And Proline Metabolism
KEGG_Hypertrophic Cardiomyopathy
KEGG_Systemic Lupus Erythematosus
KEGG_Glycosaminoglycan Degradation
KEGG_Arrhythmogenic Right Ventricular Cardiomyopathy
KEGG_Vibrio Cholerae Infection
KEGG_Hematopoietic Cell Lineage
KEGG_Biosynthesis Of Unsaturated Fatty Acids
KEGG_Glycolysis Gluconeogenesis
KEGG_Citrate Cycle TCA Cycle
KEGG_Galactose Metabolism
KEGG_Antigen Processing And Presentation
KEGG_Glyoxylate And Dicarboxylate Metabolism
KEGG_Metabolism Of Xenobiotics By Cytochrome P450
KEGG_Viral Myocarditis
KEGG_Drug Metabolism Cytochrome P450
KEGG_Fatty Acid Metabolism
KEGG_Glycosylphosphatidylinositol GPI Anchor Biosynthesis
KEGG_Protein Export
KEGG_Propanoate Metabolism
KEGG_Beta Alanine Metabolism
KEGG_Leukocyte Transendothelial Migration
KEGG_Butanoate Metabolism
KEGG_Pyruvate Metabolism
KEGG_Pentose Phosphate Pathway
KEGG_Arachidonic Acid Metabolism

Supplementary Table 2: Pathways down-regulated by doxorubicin treatment

Pathway
KEGG_Ubiquitin Mediated Proteolysis
KEGG_RNA degradation
KEGG_Spliceosome
KEGG_Wnt Signaling Pathway
KEGG_Prostate Cancer
KEGG_MAPK Signaling Pathway

Supplementary Table 3: List of Realtime PCR Primers Used

Gene name	Direction	Primer sequence
MYH7	Forward	CTCGCCAGAATGGAGTACAAA
MYH7	Reverse	CTTCATCCAGGGCCAATTCT
NKX2.5	Forward	CTACGGTTATAACGCCTACCC
NKX2.5	Reverse	CGAAGTTCACGAAGTTGTTGTT
cTnT	Forward	CAAAGGAGGCTGAAGATGGC
cTnT	Reverse	CAAAGTGAGCCTCGATCAGC
β actin	Forward	GGCATGGGTCAGAAGGATTC
β actin	Reverse	CACACGCAGCTCATTGTAGAAG
hOCT4	Forward	TCTTTCCACCAGGCCCCCGGCTC
hOCT4	Reverse	TGCGGGCGGACATGGGGAGATCC
Nanog	Forward	CATGAGTGTGGATCCAGCTTG
Nanog	Reverse	CCTGAATAAGCAGATCCATGG
hTERT	Forward	TGCGGCCGATTGTGAAC
hTERT	Reverse	CCTCTTTTCTCTGCGGAACGT
